# Chromatin remodelers HELLS, WDHD1 and BAZ1A are dynamically expressed during mouse spermatogenesis

**DOI:** 10.1530/REP-22-0240

**Published:** 2022-10-04

**Authors:** Ram Prakash Yadav, Sini Leskinen, Lin Ma, Juho-Antti Mäkelä, Noora Kotaja

**Affiliations:** 1 Institute of Biomedicine, Integrative Physiology and Pharmacology Unit, University of Turku, Turku, Finland

## Abstract

**In brief:**

Proper regulation of heterochromatin is critical for spermatogenesis. This study reveals the dynamic localization patterns of distinct chromatin regulators during spermatogenesis and disrupted sex chromatin status in spermatocytes in the absence of DICER.

**Abstract:**

Heterochromatin is dynamically formed and organized in differentiating male germ cells, and its proper regulation is a prerequisite for normal spermatogenesis. While heterochromatin is generally transcriptionally silent, we have previously shown that major satellite repeat (MSR) DNA in the pericentric heterochromatin (PCH) is transcribed during spermatogenesis. We have also shown that DICER associates with PCH and is involved in the regulation of MSR-derived transcripts. To shed light on the heterochromatin regulation in the male germline, we studied the expression, localization and heterochromatin association of selected testis-enriched chromatin regulators in the mouse testis. Our results show that HELLS, WDHD1 and BAZ1A are dynamically expressed during spermatogenesis. They display limited overlap in expression, suggesting involvement in distinct heterochromatin-associated processes at different steps of differentiation. We also show that HELLS and BAZ1A interact with DICER and MSR chromatin. Interestingly, deletion of *Dicer1* affects the sex chromosome heterochromatin status in late pachytene spermatocytes, as demonstrated by mislocalization of Polycomb protein family member SCML1 to the sex body. These data substantiate the importance of dynamic heterochromatin regulation during spermatogenesis and emphasize the key role of DICER in the maintenance of chromatin status in meiotic male germ cells.

## Introduction

The genomes of eukaryotic organisms are structurally subdivided into tightly packed heterochromatin and more loosely packed euchromatin. This structural organization also has functional consequences: while euchromatin is considered active and open for transcription, heterochromatin abounds with silencing histone modifications, such as histone H3 trimethylation on lysine 9 (H3K9me3) and DNA methylation (Saksouk *et al.* 2015). Heterochromatin can be further partitioned into constitutive heterochromatin, which is a major component of eukaryotic genomes, and facultative heterochromatin, which is dynamically formed and regulated during development and lineage-specific differentiation (Janssen *et al.* 2018). Constitutive heterochromatin can be found at many locations along the chromosomes, most pronouncedly at areas near the centromeres, that is pericentric heterochromatin (PCH) (Saksouk *et al.* 2015). It is mainly composed of repeat sequences emphasizing the need to keep it transcriptionally silent. PCH is enriched with non-coding tandem repetitions called major satellite repeats (MSR). Despite accumulation of repressive chromatin marks, MSR chromatin is transcribed during cellular stress and differentiation, early embryonic development, in cancer and spermatogenesis (Eymery *et al.* 2009, Probst *et al.* 2010, Ting *et al.* 2011, Casanova *et al.* 2013, Biscotti *et al.* 2015, Trofimova *et al.* 2015, Yadav *et al.* 2020).

Heterochromatin is dynamically re-organized and regulated during spermatogenesis. Male germline stem cells (GSCs) contain a relatively low amount of microscopically visible heterochromatin, consistent with less abundant heterochromatin marks in stem cells when compared to differentiated cells (Meshorer *et al.* 2006). There is nonetheless compelling evidence that epigenetic regulation of male GSCs is essential for their lifelong maintenance (reviewed in (Mäkelä & Hobbs 2019)). In mitotic spermatogonia, the amount of heterochromatin gradually increases as they progress from type A to Intermediate to type B spermatogonia. During meiosis, heterochromatin has important functions in controlling chromosomal stability, homolog pairing, centromere clustering and synapsis, thus promoting fidelity of meiosis (Peters *et al.* 2001, Seandel *et al.* 2007, Tachibana *et al.* 2007, Takada *et al.* 2011, Scherthan *et al.* 2014). Meiotic spermatocytes are characterized by several chromocenters and the sex chromosome-containing sex body (Perry *et al.* 2001, Turner 2007). The sex body forms when X and Y chromosomes undergo DNA damage response pathway-dependent meiotic sex chromosome inactivation that silences transcription (Alavattam *et al.* 2021). Before the meiotic divisions, the sex body disintegrates, but in spermatids, postmeiotic sex chromatin (PMSC) can be observed as a separate heterochromatic domain adjoining the single prominent chromocenter (Namekawa *et al.* 2006). The chromocenter in spermatids is thought to facilitate the ordered compaction of the haploid genome in elongating spermatids (Haaf & Ward 1995, Meyer-Ficca *et al.* 1998).

We have previously shown that, despite accumulation of silencing histone modifications, such as trimethylations of lysine 20 of histone H4 (H4K20me3) and H3K9me3 (Almouzni & Probst 2011), PCH is expressed during spermatogenesis and produces MSR transcripts (Yadav *et al.* 2020). We have also shown that the endonuclease DICER associates with PCH and SET-domain containing methyltransferases SETDB1 and SUV39H2 (Suppressor of variegation 3–9 homolog 2) in male germ cells (Yadav *et al.* 2020) and processes MSR transcripts. Our data support a mechanism where MSR transcripts processed by DICER are needed to recruit SETDB1 and SUV39H2 to PCH for heterochromatin maintenance (Johnson *et al.* 2017, Velazquez Camacho *et al.* 2017) and faithful completion of meiosis. Given the fact that DICER forms multiprotein complexes to complete its diverse functions, a deeper understanding of this process requires more detailed characterization of other factors involved.

In addition to histone and DNA modifiers, several factors have been implicated in the regulation of PCH (Saksouk *et al.* 2015, Nishibuchi & Déjardin 2017). For example, HELLS (Helicase, lymphoid-specific, LSH) is a chromatin remodeling protein that is critical for constitutive heterochromatin regulation and is especially closely associated with PCH (Muegge 2005). It facilitates DNA methylation at repeat sequences also elsewhere in the genome, contributing to the maintenance of these loci in a transcriptionally quiescent state (Huang *et al.* 2004, Dunican *et al.* 2013, Yu *et al.* 2014, Ren *et al.* 2015). WDHD1 (WD repeat and HMG-box DNA-binding protein 1) is a nucleoplasmic DNA-binding protein (Köhler *et al.* 1997) associated with regulation of the cell cycle, DNA replication and repair (Hao *et al.* 2015, Villa *et al.* 2016, Abe *et al.* 2018) and cellular response to DNA damage (Chen *et al.* 2017, Li *et al.* 2017). Hsieh *et al.* (2011) also suggest that DICER interaction with MSR transcripts might be at least partially dependent on interaction with WDHD1 (Hsieh *et al.* 2011), placing WDHD1 in the focus of DICER’s PCH-associated function.

BAZ1A (Bromodomain Adjacent To Zinc Finger Domain 1A; also known as ACF1) is the key subunit of chromatin remodeler complexes ACF (ATP-dependent Chromatin assembly and remodeling Factor) and CHRAC (CHRomatin Accessibility Complex). These complexes assemble/disassemble and restructure nucleosomes, thus blocking or granting access to other proteins active in DNA transcription, replication or repair (Eberharter *et al.* 2001, He *et al.* 2008, Lan *et al.* 2010, Sánchez-Molina *et al.* 2011, Aydin *et al.* 2014, Morgan *et al.* 2020). Interestingly, BAZ1A is also involved in enabling replication of condensed chromatin at PCH (Collins *et al.* 2002). Finally, SCML2 (Scm Polycomb Group Protein Like 2), a germline-specific subunit of Polycomb repressive complex 1 (PRC1), controls the timing of expression of spermatogenic genes and suppresses somatic gene expression in the male germline (Hasegawa *et al.* 2015, Maezawa *et al.* 2017, 2018*a*). SCML2 specifically accumulates on PCH, where it coordinates the modification of histone residues during meiosis (Maezawa *et al.* 2018*b*). In spermatids, SCML2-deficiency results in disorganization of heterochromatin and formation of ectopic patches of facultative heterochromatin (Maezawa *et al.* 2018*b*) highlighting the central role of SCML2 in heterochromatin regulation.

Despite the critical role of heterochromatin in the genome organization and function, the molecular players involved in its formation and regulation in the male germline are still poorly described. To this end, we aimed at characterizing the cell type-specific localization and function of HELLS, WDHD1 and BAZ1A in spermatogenic cells. Based on our previous data, we specifically focused on their plausible association with DICER and MSR chromatin. Our data indicate that HELLS, WDHD1 and BAZ1A are dynamically regulated during spermatogenesis, and HELLS and BAZ1A associate with DICER and are intimately involved in PCH regulation in male germ cells. We also show that the sex chromatin-associated heterochromatin regulation is compromised in the absence of DICER, further highlighting the role of DICER in heterochromatin regulation in the male germline.

## Materials and methods

### Animals

Mice were maintained in the Central Animal Laboratory of University of Turku, Turku, Finland. They were kept at 12 h/12 h light/darkness cycle, in a humidity and temperature-controlled environment in individually-ventilated cages (Tecniplast, Buguggiate, Italy). They had free access to food and water. Germ cell-specific conditional *Dicer1* knockout (*Dicer1* cKO) mice were generated using *Neurogenin3* (*Ngn3*) promoter-driven *Cre* recombinase expression in mice carrying a floxed *Dicer1* allele, as previously described (Korhonen *et al.* 2011), resulting in *Dicer1* deletion in all differentiation-primed germ cells. The mice were of a mixed genetic background (C57BL/6J and Sv129). WT littermates were used as controls. WT mice from C57BL/6J or C57BL/6N strains were used for immunoprecipitation experiments and whole-mount stainings. Animal husbandry and use were carried out according to Finnish laws and following the guidelines of Ethics of Animal Experimentation at University of Turku in accordance with the Guide for Care and Use of Laboratory Animals. The use of experimental animals in this study was approved by University of Turku Ethics Committee for animal experiments.

### Immunofluorescence and fluorescence signal colocalization analysis

For immunofluorescence (IF), the testes from adult *Dicer1* cKO and WT mice were collected and fixed with 4% PFA (paraformaldehyde) overnight, followed by dehydration and embedding into paraffin. Five-µm-thick sections were cut and processed for IF staining as previously described (Yadav *et al.* 2020). Briefly, following rehydration and antigen retrieval, quenching, permeabilization and blocking (2% normal donkey serum (NDS) + 2% bovine serum albumin (BSA) + 0.1% Triton X-100 in PBS), the samples were incubated overnight at +4°C with following primary antibodies: HELLS (1:500, Millipore, ABD41), WDHD1 (1:500, A301-141A-T), phospho-Histone H2A.X (Ser139) (1:500, Millipore, 05-636), HP1β (1:25; Millipore, MAB3348), BAZ1A (1:500, Sigma, HPA002730), SCML1 (1:100, Santa Cruz, sc-135622) and SCML2 (DSHB Hybridoma Product PCRP-SCML2-1D12 that was deposited to the DSHB by Common Fund – Protein Capture Reagents Program). After washes, the samples were incubated for 60 min at RT with appropriate secondary antibodies (A-21202, A-21203, A-21206, A-21207 and A-21208, all purchased from Invitrogen and diluted 1:1000). All antibody dilutions were prepared in the blocking solution. Finally, the samples were stained for 10 min at RT with DAPI and mounted with ProLong Diamond Antifade mountant (Invitrogen). Images were captured using laser scanning confocal microscope (Zeiss LSM780) or 3i CSU-W1 spinning disk confocal microscopy (3i Intelligent Imaging Innovations; Denver, CO, USA). The epithelial stages of seminiferous tubule cross-sections were determined according to (Meistrich & Hess 2013, Mäkelä *et al.* 2020). To study the colocalization of BAZ1A and SCML1, combined IF, followed by Z-stacks of the WT and cKO testis sections were captured using 3i CSU-W1 spinning disk confocal microscope. All the Z- stacks were merged to make a single image and colocalization analysis was performed using ImageJ. Signal colocalization in stage IX–X WT (*n* = 129) and *Dicer1* cKO (*n* = 133) sex bodies was manually quantified from two independent immunostanings per genotype.

### Whole-mount immunostaining and A_undiff_ quantitation

Testes from three adult WT mice were dissected, decapsulated, fixed with 4% PFA (2–6 h at RT) and processed for whole-mount immunostainings as previously described (Mäkelä *et al.* 2020). The tubules were incubated overnight with the following antibodies: HELLS (1:200, Millipore, ABD41), WDHD1 (1:200, A301-141A-T), GFRa1 (AF560, R&D Systems, 1:250) and SOX3 (AF2569, R&D Systems, 1:200) and DNMT3A (IMG-268A, Imgenex/Novus, 1:200). Following washes, the tubules were incubated with corresponding secondary antibodies (ThermoFisher, A31571, A11055 and A10040; all diluted 1:500). Finally, the tubules were poured onto a microscope slide, ordered into linear strips and mounted using ProLong Diamond Antifade mountant with DAPI (Invitrogen). The stainings were imaged using 3i Spinning disk confocal microscope.

In HELLS/GFRa1 and WDHD1/GFRa1 double-stainings, GFRa1-positive type A-undifferentiated (A_undiff_) spermatogonia were divided into three categories, A-single (A_s_, singly isolated cells), A-paired (A_pr_, two interconnected cells) and A-aligned-4 (A_al4_, four interconnected cells). These cells are considered to contain enriched stem cell activities in the male germline (Hara *et al.* 2014, Mäkelä & Hobbs 2019). HELLS or WDHD1 signal intensities in these GFRa1-positive cells were assessed as high (comparable to signal strength in differentiating spermatogonia (A_diff_) that were uniformly positive for both), low (clearly lower than in A_diff_) or negative (no signal). The quantification was performed systematically on random fragments of adult mouse seminiferous tubules. For HELLS and WDHD1, in total 1391 and 1245 GFRa1-positive cells, respectively, were quantified (*n* = 3, average 465 ± 108 and 414 ± 108, respectively). SOX3-positive/DNMT3A-low/negative cells (differentiation-primed progenitor A_undiff_ spermatogonia (McAninch *et al.* 2020)) were also assessed for HELLS and WDHD1 positivity. Altogether 1196 and 889 SOX3-positive cells were quantified for HELLS and WDHD1 staining, respectively (*n* = 2-3, average 598 ± 57 and 296 ± 19, respectively). The data were normalized by calculating the percentage of cells that fell into each category.

### Chromatin immunoprecipitation

Germ cells from 18-day-old mouse testes were released by enzymatic digestion with collagenase type I (Worthington) in 0.1% glucose in PBS for 60–70 min at RT. This time point was selected due to high level of DICER expression and enrichment of MSR transcripts at 18 days of age (Korhonen *et al.* 2015, Yadav *et al.* 2020). Cell suspension was filtered through a 100-µm cell strainer and centrifuged at 500 ***g*** for 5 min. Pellets were then re-suspended in ice-cold 0.1% glucose in PBS and passed through a 40-µm cell strainer. Then pellets were re-suspended in 10 mL of PBS and cross-linked in 1% PFA for 20 min at RT. Glycine (125 mM) was added for 5 min at RT to stop cross-linking, and cells were pelleted at 500 ***g*** for 10 min. Chromatin immunoprecipitation (ChIP) assay was performed, as previously described (Yadav *et al.* 2020). Lysates were incubated with the following antibodies: HELLS (Millipore, ABD41), WDHD1 (BioLegend, 630301, clone 20G10), BAZ1A (Sigma, HPA002730), DICER (Bethyl, A301-936A), RNA polymerase II (A300-653A-M) plus its phosphorylated form S2 (A300-654A-T) and IgG ctrls (Santa Cruz, SC-2025 and Neomarkers, NC100-P1).

DNA was isolated using TRIsure (Bioline) and 2-propanol precipitation. PCR was performed using the primer sequences and annealing temperatures specified in Table 1.

**Table 1:** Primer sequences and annealing temperatures of the studied mouse templates.

Target	Forward primer	Reverse primer	T_ann_ (°C)
*MajorSR*	5′-GACGACTTGAAAAATGACGAAATC-3′	5′-CATATTCCAGGTCCTTCAGTGTGC-3′	57
*MinorSR*	5′-CATGGAAAATGATAAAAACC-3′	5′-CATCTAATATGTTCTACAGTGTGG-3′	57
*Line1*	5′-TTTGGGACACAATGAAAGCA-3′	5′-CTGCCGTCTACTCCTCTTGG-3′	60
*SineB1*	5′-GTGGCGCACGCCTTTAATC-3′	5′-GACAGGGTTTCTCTGTGTAG-3′	60
*SineB2*	5′-GAGATGGCTCAGTGGTTAAG-3′	5′-CTGTCTTCAGACACTCCAG-3′	60
*IAP*	5′-AGCAGGTGAAGCCACTG-3′	5′-CTTGCCACACTTAGAGC-3′	62
*Gapdh*	5′-AGTGCCAGCCTCGTCCCGTA-3′	5′-AGGCGCCCAATACGGCCAAA-3′	57
*18S*	5′-GTAGTCGCCGTGCCTACCAT-3′	5′-TTTTCGTCACTACCTCCCCG-3′	60

*18S*, 18S ribosomal DNA; *Gapdh*, glyceraldehyde 3-phosphate dehydrogenase; *IAP*, intracisternal A particle; *Line1*, long interspersed nuclear element 1; *Sine B1*, short interspersed nuclear element B1; SR, satellite repeat; T_ann_, annealing temperature.

### Immunoprecipitation and Western blotting

Testes from 18-day-old mice were decapsulated and lysed in a lysis buffer as previously described (Yadav *et al.* 2020). Following lysis, Dynabeads Protein G (Invitrogen) precleared (1 h at +4°C) lysates were incubated with 2–3 µg of the following antibodies overnight in rotation at +4°C: HELLS (Millipore, ABD41), WDHD1 (BioLegend, 630301, clone 20G10), BAZ1A (Sigma, HPA002730), YY1 (Bethyl, A302-779A) and IgG ctrls (Santa Cruz, SC-2025 and Neomarkers, NC100-P1). On the following day, the samples were incubated with pre-blocked (5% BSA in PBS) Dynabeads Protein G (Invitrogen) for 2 h and then washed thrice with a buffer containing 50 mM Tris–HCl pH 7.5, 150 mM NaCl, 2 mM MgCl_2_, 0.2% Triton X-100 and 0.2% NP-40, followed by three washes in a buffer containing 50 mM Tris–HCl pH 7.5, 150 mM NaCl, 2 mM MgCl_2_, 0.1% Triton X-100 and 0.1% NP-40. Protein complexes were finally eluted in Laemmli buffer by incubating at 90°C for 10 min. For Western blotting, proteins were separated on a 4–20% polyacrylamide gel (Mini-PROTEAN, Bio-Rad) and transferred onto a PVDF membrane overnight on ice at 60 V. To verify the success of immunoprecipitation and identify the co-immunoprecipitated proteins, PVDF membranes were incubated with primary antibodies: HELLS (Millipore, ABD41), WDHD1 (BioLegend, 630301, clone 20G10), BAZ1A (Sigma, HPA002730), YY1 (Bethyl, A302-779A), DICER (Sigma, SAB4200087), SCML1 (Santa Cruz, sc-135622) and IgG ctrls and secondary antibodies (anti-rabbit HRP-conjugated, 1:1000, CST, 7074S; anti-mouse HRP-conjugated, 1:1000, CST, 7076S; Anti-rabbit light chain HRP-conjugated, 1:1000, Millipore, MAB201P) diluted in 4% skimmed milk powder in TBST (0.05%) for 1 h at RT. The signals were visualized by western lightning ECL Pro (NEL122001EA, PerkinElmer) reagent and ImageQuant LAS 4000 Biomolecular Imager (GE Healthcare).

### Reanalysis of single-cell RNA-seq data and statistical methods

To visualize the expression of genes of interest in adult male mouse germ cells, we took advantage of previously published adult mouse testis scRNA-seq datasets (Hermann *et al.* 2018). Loupe Cell Browser v6.0.0 from 10x Genomics was used to produce tSNE (t-distributed stochastic neighbor embedding) plots to visualize the expression of *Hells* and *Wdhd1* in spermatogonial subsets (adult mouse-sorted spermatogonia dataset). Loupe Cell Browser v6.2.0 was used to produce heat maps to visualize gene expression during spermatogenesis in specific germ cell subsets (mouse unselected spermatogenic cells dataset – downloaded from Mendeley data – doi: 10.17632/kxd5f8vpt4.1). Median Normalized Average values of genes without somatic cells were first exported. Then six genes were searched (*Dicer1, Hells, Wdhd1, Scml2, Baz1a* and* Scml1*) from this dataset to examine their expression trends during spermatogenesis. The statistical analyses were performed with GraphPad Prism 8 software (GraphPad Software, La Jolla, CA, USA). The numerical data were analyzed for statistically significant differences using two-tailed *t*-test for pairwise comparisons and Fisher’s exact test to determine if there was a significant association between groups. *P* values < 0.05 were considered statistically significant.

## Results

### HELLS and WDHD1 are expressed in spermatogonia and spermatocytes

**Figure 1:**
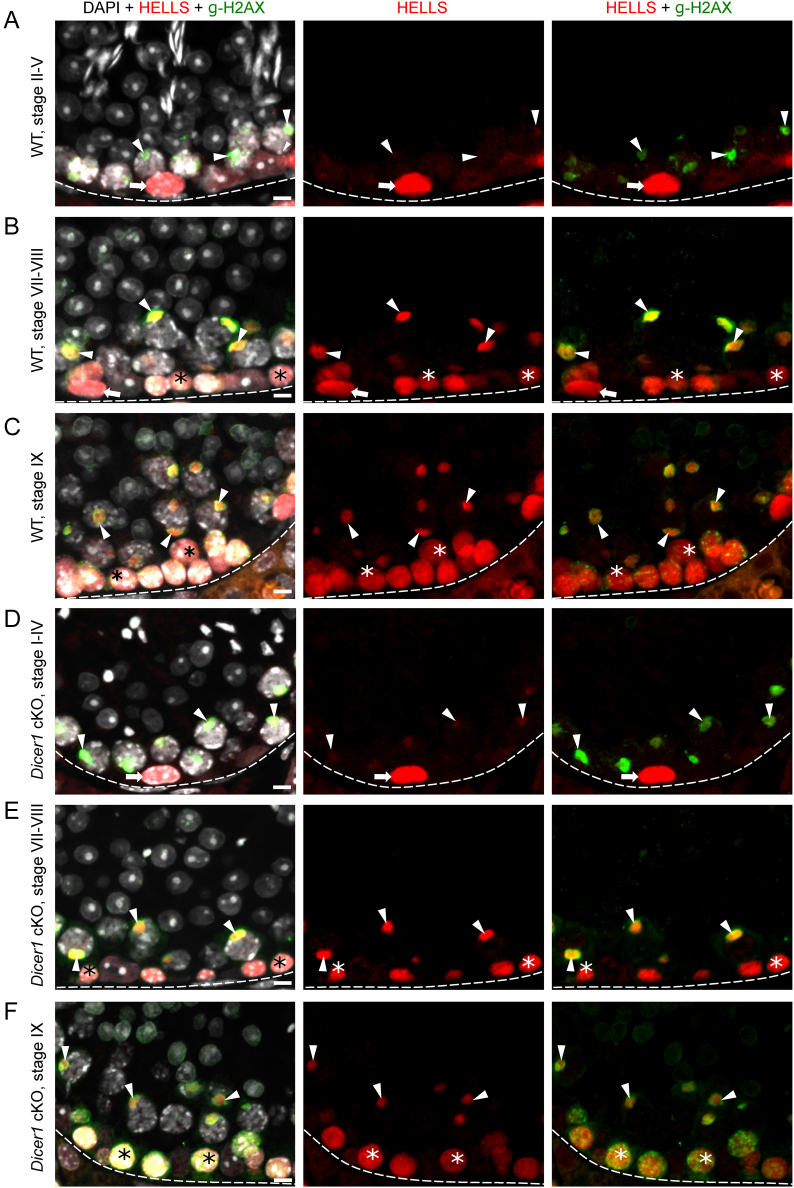
HELLS is expressed by spermatogonia and spermatocytes in WT and *Dicer1* cKO mice. (A) In stage II–V of WT mouse seminiferous epithelial cycle, HELLS (red) expression is restricted to spermatogonia (arrow). Sex bodies (arrowhead) in early-pachytene spermatocytes are positive for γH2AX (green). (B) In WT stages VII–VIII, HELLS is expressed in spermatogonia (arrow), preleptotene spermatocytes (asterisk) and mid-pachytene spermatocytes, where the signal concentrates to the γH2AX-positive sex bodies (arrowhead). (C) In WT stage IX a strong HELLS expression is maintained in leptotene spermatocytes (asterisk) and sex bodies (arrowhead) of late-pachytene spermatocytes. In *Dicer1* cKO mice the expression pattern of HELLS is similar to WT in stages (D) I–IV, (E) VII–VIII and (F) IX. DAPI stains chromatin (white). Basement membrane of seminiferous epithelium (dotted line), spermatogonia (arrow), early spermatocytes (asterisk), sex body (arrowhead). Scale bars 10 µm.

To study the expression pattern of chromatin remodelers of interest, we performed immunofluorescent labeling of adult mouse testis cross-sections and assessed the expression pattern of HELLS, WDHD1 and BAZ1A in a stage-dependent manner. HELLS was found to be strongly expressed in spermatogonia plus preleptotene and leptotene spermatocytes (Fig. 1 and Supplementary Fig. 1, see section on supplementary materials given at the end of this article). In early pachytene spermatocytes, HELLS expression was weak (Fig. 1A), but a strong signal appeared in mid-to-late pachytene spermatocytes, where HELLS was localized to a distinct nuclear domain (Fig. 1B and C). Based on double-labeling with a γH2AX antibody, this HELLS-positive nuclear structure was identified as the sex body composed of heterochromatinized sex chromosomes (Turner 2007) (Fig. 1B and C). Post-meiotic spermatids were devoid of HELLS expression.

**Figure 2:**
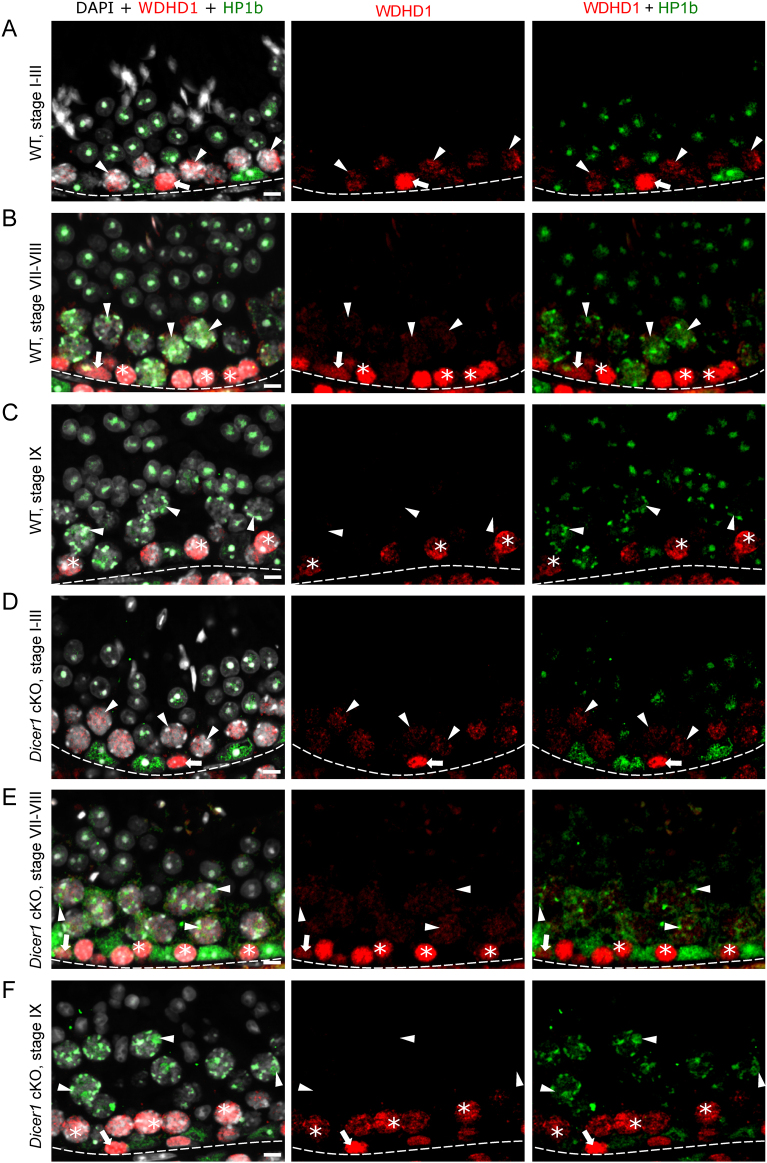
WDHD1 is expressed by spermatogonia and early spermatocytes in WT and *Dicer1* cKO mice. (A) In stage I–III of WT mouse seminiferous epithelial cycle, WDHD1 (red) is highly expressed in spermatogonia (arrow), whereas the expression level in early-pachytene spermatocytes (arrowhead) is low. HP1β (green) stains heterochromatin. (B) In WT stages VII–VIII, WDHD1 is expressed by spermatogonia (arrow) and preleptotene spermatocytes (asterisk). In mid-pachytene spermatocytes (arrowhead), the WDHD1 expression level is low. (C) By WT stage IX, late-pachytene spermatocytes (arrowhead) become negative for WDHD1. WDHD1 is also downregulated upon preleptotene-to-leptotene (asterisk) transition. In *Dicer1* cKO mice, the expression pattern of WDHD1 is similar to WT in stages (D) I–III, (E) VII–VIII and (F) IX. DAPI stains chromatin (white). Basement membrane of seminiferous epithelium (dotted line), spermatogonia (arrow), early spermatocytes (asterisk), pachytene spermatocytes (arrowhead). Scale bars 10 µm.

Similar to HELLS, WDHD1 was also strongly expressed in spermatogonia and early spermatocytes (Fig. 2A, B and C). However, WDHD1 levels clearly started to decline in leptotene spermatocytes (Fig. 2C). Early-to-mid pachytene spermatocytes still expressed low levels of WDHD1, but the signal was absent from stage IX pachytene spermatocytes onwards. In early-to-mid primary spermatocytes, WDHD1 had a nuclear granular localization pattern but the signal did not overlap with the DAPI-dense foci or with HP1β-positive foci, suggesting that WDHD1 signal is not specifically associated with heterochromatin in meiotic germ cells (Fig. 2A, B and C). Furthermore, in contrast to HELLS, no sex body accumulation was observed.

**Figure 3:**
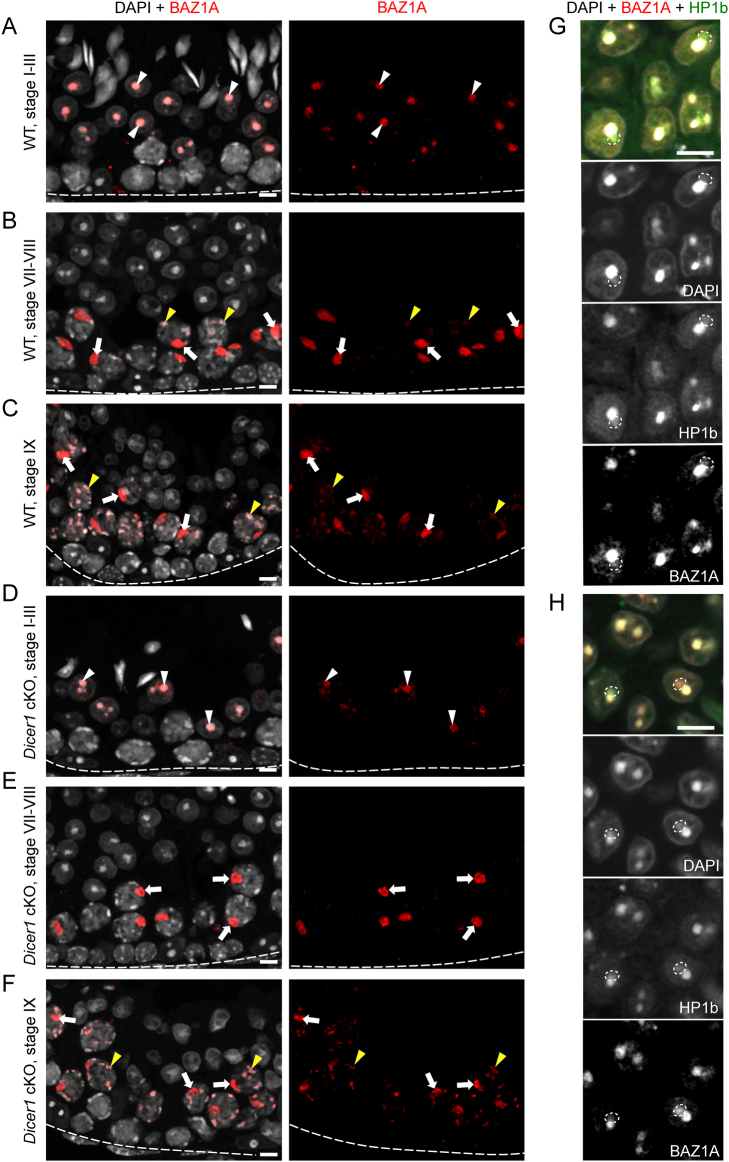
BAZ1A localizes to mid-to-late sex bodies. (A) In stage I–III of WT mouse seminiferous epithelial cycle BAZ1A (red) localizes to the chromocenter (white arrowhead) of step 1–3 round spermatids. (B) By WT stages VII–VIII, BAZ1A becomes absent from round spermatids but is found highly accumulated in the sex body of pachytene spermatocytes (arrow). Other heterochromatic foci (yellow arrowheads) in stages VII–VIII pachytene spermatocytes also contain BAZ1A although less than the sex bodies. (C) In WT stage IX, the staining pattern is similar to stages VII–VIII. (D) In *Dicer1* cKO mice the expression pattern of WDHD1 is similar to WT in stages (D) I–III, (E) VII–VIII and (F) IX. Low levels of BAZ1A are also seen in DAPI/HP1β-positive PMSC (marked with dotted ovals) both in (G) WT and (H) *Dicer 1* cKO early round spermatids. DAPI stains chromatin (white). Basement membrane of seminiferous epithelium (dotted line), sex body (arrow), chromocenter (white arrowhead), heterochromatic foci (yellow arrowhead), PMSC (dotted oval). Scale bars 10 µm.

The strong expression of HELLS and WDHD1 in early phases of spermatogenesis was different from that of BAZ1A, whose expression has earlier been shown to be induced only later, in mid-pachytene spermatocytes (Dowdle *et al.* 2013). Here, we also showed that the expression of BAZ1A is absent from spermatogonia and spermatocytes up to the early pachytene phase (Fig. 3A). In mid-to-late pachytene spermatocytes, BAZ1A localization enriched in nuclear foci, that have previously been shown to correspond to the sex body (Dowdle *et al.* 2013) (Fig. 3B and C). In addition, we observed a weaker BAZ1A signal in DAPI-dense heterochromatin foci (Fig. 3B and C). Strong association of BAZ1A with the heterochromatin was sustained in early (I–V) round spermatids where it was found enriched in the chromocenter (Fig. 3A). Low levels of BAZ1A were also observed in PMSC (Fig. 3G) in early round spermatids. No BAZ1A signal was detected in late round spermatids or elongating spermatids (Fig. 3B and C).

### WDHD1 is upregulated upon differentiation commitment in the male germline

**Figure 4:**
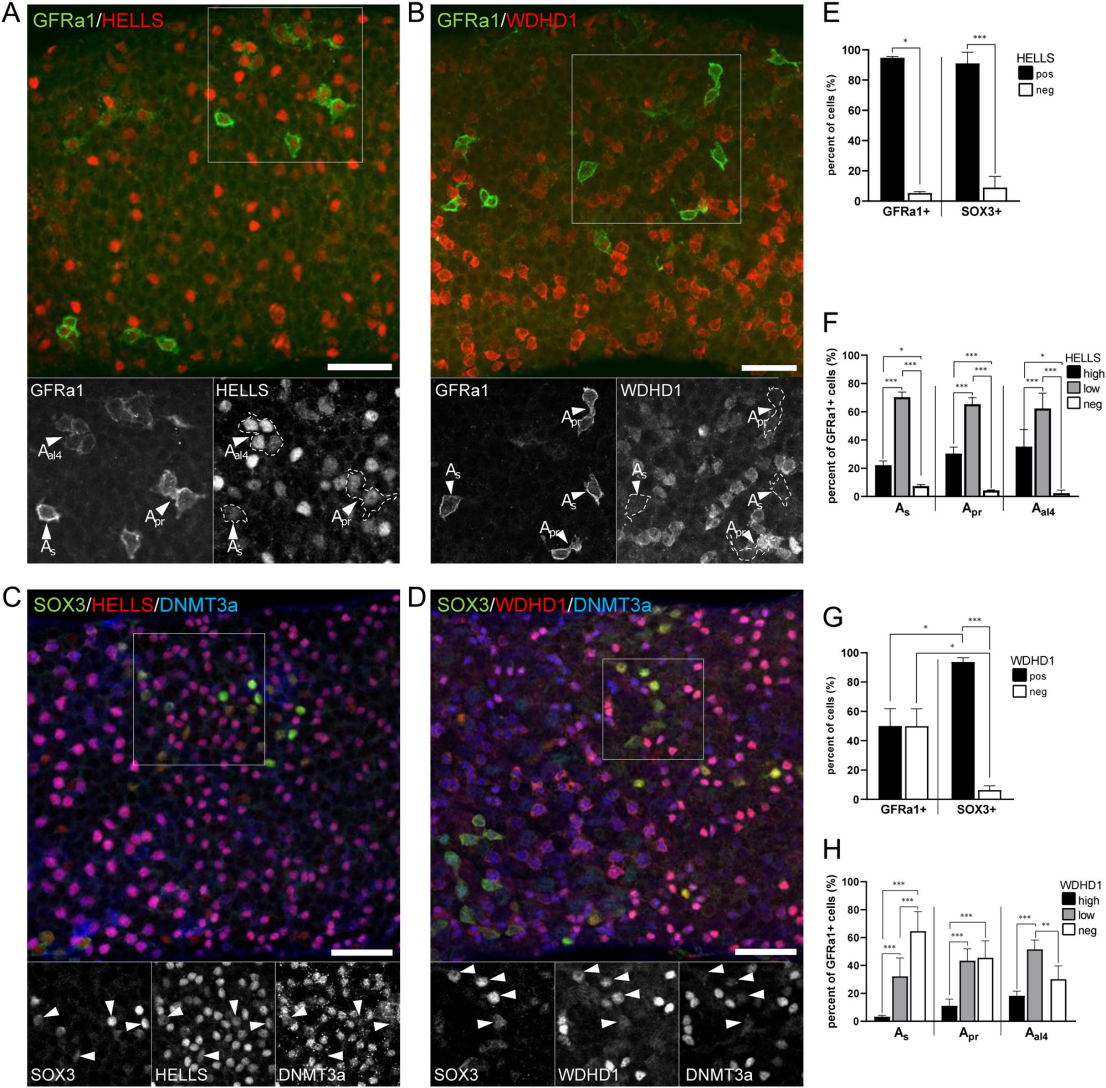
WDHD1 is upregulated upon spermatogenic differentiation commitment. Both (A) HELLS (red) and (B) WDHD1 (red) display a heterogeneous expression in GFRa1-positive (green) stem A_undiff_ spermatogonia. Grayscale insets show the area indicated by white boxes separately for each channel. Arrowheads point at A_s_, A_pr_ and A_al4_ GFRa1-positive spermatogonia. (C) In SOX3-positive (green)/DNMT3A-negative/low (blue) progenitor A_undiff_ spermatogonia, HELLS (red) and (D) WDHD1 (red) are expressed in a more uniform manner. DNMT3A-positive (blue) A_diff_ also express both HELLS and WDHD1. Arrowheads (in grayscale insets) point at SOX3-positive/DNMT3A-negative/low progenitor spermatogonia. Scale bars 50 µm. Quantitation of (E) HELLS-positive and (G) WDHD1-positive cells in GFRa1-positive and SOX3-positive A_undiff_ subsets. As A_undiff_ progress A_s_ -> A_pr_ -> A_al4_ there is a gradual upregulation of (F) HELLS and (H) WDHD1 expression and the number of HELLS and WDHD1-negative cells consistently decreases. Statistical significancies were studied using *t*-test and Fisher’s exact test; ***P < 0.001; **P < 0.01; *P < 0.05.

Given the prominent expression of HELLS and WDHD1 in spermatogonia (Figs 1, 2 and Supplementary Fig. 1), we wanted to study their expression pattern during spermatogonial differentiation in more detail. Transition from undifferentiated (A_undiff_) to differentiating spermatogonia (A_diff_) represents a remarkable developmental and epigenetic shift in the male germline. While stem cell capacity is thought to reside in all or a fraction of A_undiff_, A_diff_ are irreversibly committed to spermatogenesis (Mäkelä & Hobbs 2019). To study if HELLS and WDHD1 expression is affected by the differentiation commitment, we performed whole-mount stainings of adult WT mouse seminiferous tubules. We showed that while DNMT3A-positive (Shirakawa *et al.* 2013) A_diff_ were uniformly positive for HELLS and WDHD1, A_undiff_ displayed a heterogeneous expression for both (Fig. 4A, B, C and D).

To study this in more detail, we analyzed co-expression of HELLS and WDHD1 with GFRa1 (GDNF family receptor alpha 1) or SOX3 (SRY-related HMG box 3), that are mutually exclusively expressed in A_undiff_ spermatogonia (McAninch *et al.* 2020). GFRa1 is expressed by A-single (A_s_), A-paired (A_pr_) and A-aligned-4 (A_al4_) A_undiff_, and the GFRa1-positive population is considered to contain the stem cells of the male germline (Mäkelä & Hobbs 2019). SOX3 staining is typically seen in longer chains of A_undiff_ (A_al4-16_); these cells are the progenitor cells that no longer undergo self-renewal under steady-state spermatogenesis but are primed for differentiation (McAninch *et al.* 2020). When HELLS expression was quantified in GFRa1-positive (A_s_, A_pr_ and A_al4_) and SOX3-positive cells, it was seen that most of these cells expressed HELLS (Fig. 4E). However, there was a tendency that the number of HELLS-negative or low cells became smaller as A_undiff_ progressed A_s_ -> A_pr_ -> A_al4_ (Fig. 4F). We also observed that WDHD1 staining intensity clearly increased when A_s_ formed first A_pr_ and then A_al4_ (Fig. 4H) indicating that WDHD1 expression inversely correlates with stemness in the male germline. These data were also supported by reanalysis of available single-cell RNA-sequencing data (Hermann *et al.* 2018) (Supplementary Fig. 2A). When studied as a bulk population of GFRa1-positive vs SOX3-positive cells, around 96% of the latter were positive for WDHD1, whereas the same figure for GFRa1-positive cells was 50% (Fig. 4G). These data demonstrate that there is a clear upregulation of spermatogonial WDHD1 expression when they transit from stem to progenitor A_undiff_ spermatogonia.

### HELLS and BAZ1A interact with pericentric heterochromatin in the mouse testis

**Figure 5:**
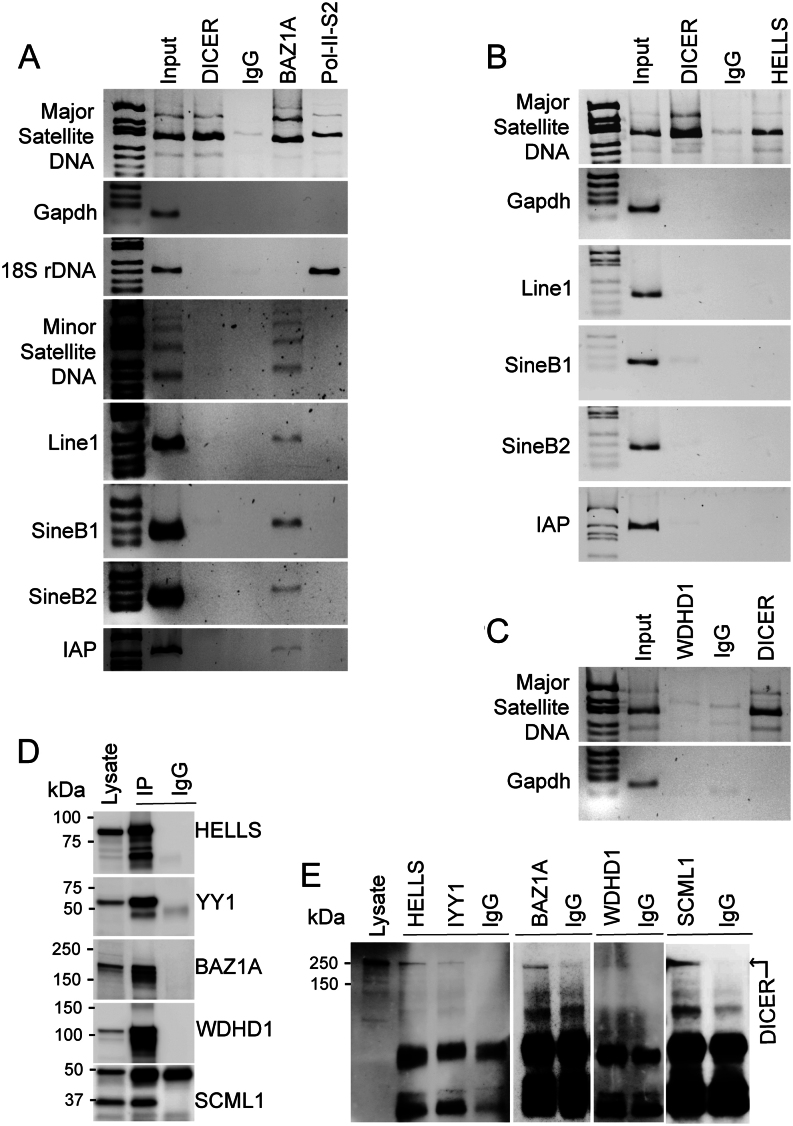
HELLS, BAZ1A and DICER interact with MSR region of the pericentric heterochromatin. (A) Chromatin immunoprecipitation using DICER, BAZ1A and Ser2-phosphorylated RNA polymerase II (Pol-II-S2) antibodies followed by detection of different chromosomal repeat regions by PCR using specific primers for major and minor satellite DNA repeats, Line1, SineB1, SineB2 and IAP transposable elements. 18S ribosomal DNA (rDNA) primers were used as a positive control for Pol-II-S2 association. (B) Chromatin immunoprecipitation using DICER and HELLS antibodies and primers for major satellite DNA repeat and different transposable elements. (C) Chromatin immunoprecipitation using DICER and WDHD1 antibodies and primers for major satellite DNA repeats. For A-C, rabbit IgG was used as a negative control for immunoprecipitation, and primers for Gapdh were used as a negative control for chromatin association. (D) Western blot images to validate the successful immunoprecipitation (IP) of HELLS (95 kDa), YY1 (60–65 kDa), BAZ1A (178 kDa) WDHD1 (126 kDa) and SCML1 (37 kDa) from the mouse testis. Western blotting was performed with the same antibodies that were used for IP. Rabbit IgG (IgG) was used as a negative control. (E) Western blotting of HELLS, YY1, BAZ1A, WDHD1 and SCML1 IPs with anti-DICER antibody (~250 kDa).

To get an insight into a possible interaction of these chromatin modifiers with the PCH, we next performed ChIP using specific antibodies and primers amplifying MSR DNA. Our data showed that both HELLS and BAZ1A interact with MSR DNA and therefore localize to the PCH (Fig. 5A and B). Previously characterized association of DICER with MSR DNA was used as a positive control. As an indication of transcriptional activity, the MSR DNA was also shown to be associated with the phosphorylated (Ser 2) RNA polymerase II, which was also associated with 18S ribosomal DNA as a positive control (Fig. 5A). The housekeeping gene *Gapdh* worked as a negative control for the association with the heterochromatin regulators. As expected from its localization pattern (Fig. 2), WDHD1 did not interact with MSR DNA in male germ cells (Fig. 5C). To better understand the role of HELLS and BAZ1A in repeat sequences’ regulation, we also looked at their occupancy at minor satellite repeat DNA and the transposable elements *Line1, Sine B1-B2* and *IAP*. Intriguingly, only BAZ1A was found to interact with the other repeat sequences, whereas HELLS specifically associated only with MSR DNA (Fig. 5A and B).

### HELLS and BAZ1A interact with DICER in the mouse testis

We have previously shown that DICER is expressed in spermatogonia, spermatocytes and spermatids, and it associates with MSR DNA and regulates MSR transcripts derived from PCH (Korhonen *et al.* 2015, Yadav *et al.* 2020). We next studied if HELLS and BAZ1A, which are also associated with heterochromatin, interact with DICER. To this end, testicular cell lysates were prepared and immunoprecipitated with HELLS, WDHD1 and BAZ1A antibodies followed by Western blotting. YY1 (Yin Yang 1), a ubiquitous and multifunctional zinc-finger transcription factor, was included in the experiment as a positive control given its previously established interaction with DICER (Zardo *et al.* 2012). Western blotting analysis showed that all antibodies were successful in immunoprecipitation and IgG controls were uniformly negative (Fig. 5D). Interestingly, we showed that HELLS and BAZ1A were found in complexes with DICER (Fig. 5E). Consistently with the absence of WDHD1 in the PCH, WDHD1 did not interact with DICER (Fig. 5E).

### Deletion of* Dicer1* affects the sex chromosome heterochromatin status

Considering the role of DICER in processing of MSR transcripts and thereby regulating heterochromatin maintenance, we then wanted to investigate if *Dicer1* deficiency affected the expression pattern or subcellular localization of HELLS, WDHD1 and BAZ1A. However, immunofluorescent analysis on *Dicer1* cKO testis sections did not reveal any obvious differences when compared to the WT control (Figs 1, 2, 3 and Supplementary Fig. 1). We also studied the localization of a germline-specific Polycomb protein SCML2 that has been found accumulated in the sex body (Hasegawa *et al.* 2015, Maezawa *et al.* 2018*b*, Menon *et al.* 2019), and it has also been demonstrated as a critical regulator of PCH in male germ cells (Maezawa *et al.* 2018*b*). A co-staining with BAZ1A revealed that SCML2 localizes to the sex body earlier (starting in stages II–IV) than BAZ1A (V–VI). Following dissociation of the sex body, SCML2 expression in spermatocytes declined and postmeiotic cells expressed very low or undetectable levels of SCML2 (Supplementary Fig. 3). Just like in the cases of HELLS, WDHD1 and BAZ1A, no differences in the expression pattern between cKO and WT control mouse testis were observed (Supplementary Fig. 3A, B, C, D, E, F, G and H). This suggests that DICER is not essential for the localization of these chromatin regulators, yet it might be crucial for their function.

**Figure 6:**
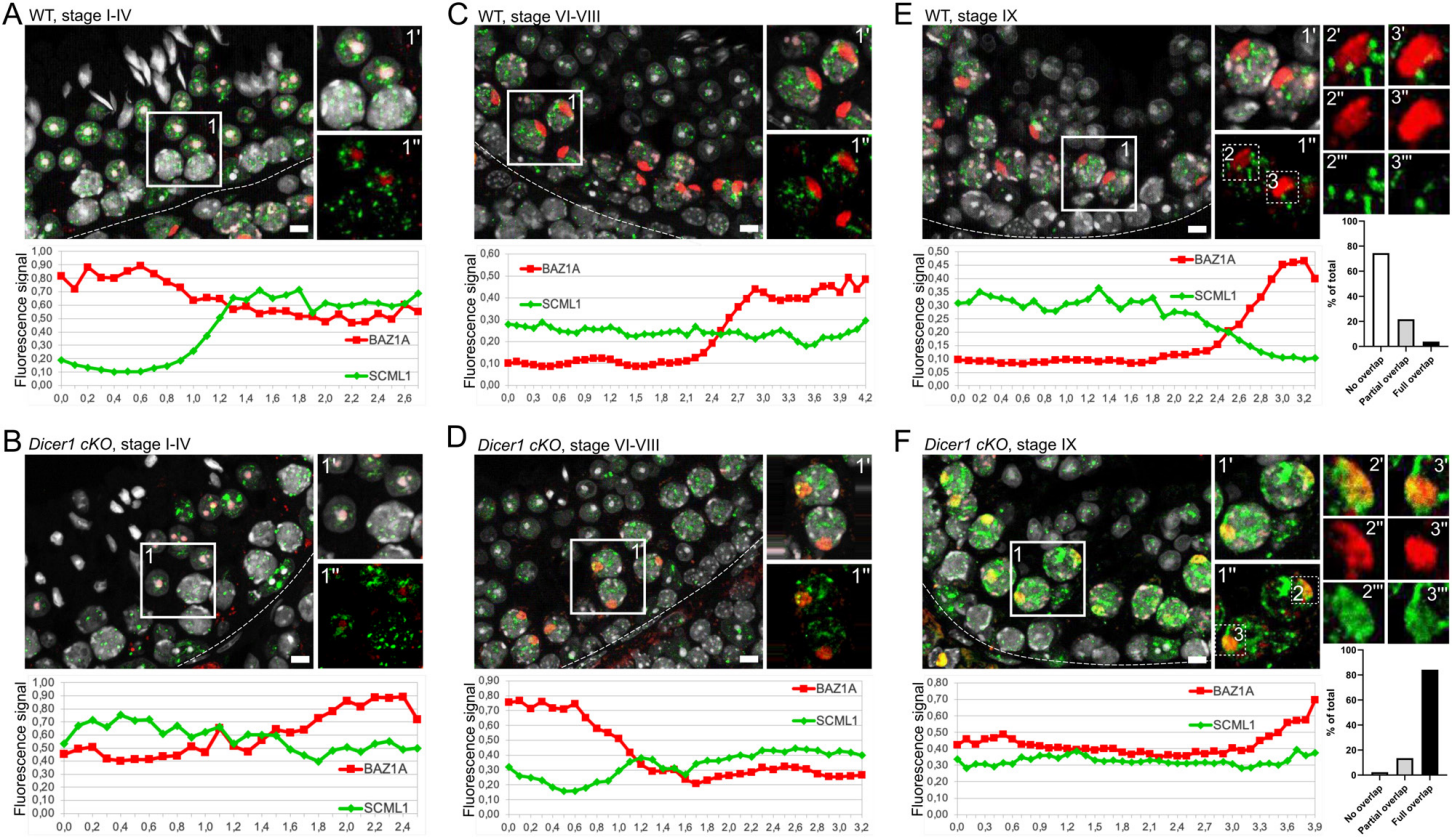
Organization of SCML1-positive foci is disrupted in *Dicer1* cKO testis. Expression and colocalization analysis of SCML1 (green) and BAZ1A (red) in stages (A) I–IV, (B) VI–VIII and (C) IX in WT testis and in stages (D) I–IV, (E) VI–VIII and (F) IX in *Dicer1* cKO testis. The Z-Stack signals of SCML1 (green) and BAZ1A (red) channels were merged to make a single image. The closer the curves (XY plot: different space points vs fluorescence signal), the better the signals co-localize in 3D space. In stages I–IV, BAZ1A localizes to chromocenter of round spermatids, whereas SCML1 is seen in DAPI-low euchromatic foci both in (A) WT and (B) cKO testis. (C) In WT stages VI–VIII, BAZ1A localizes to the sex body and heterochromatic foci in spermatocytes, while SCML1 in enriched in euchromatic foci. (D) In cKO testis, however, SCML1 signal is also seen in the sex body overlapping with BAZ1A (yellow). (E) In WT stage IX, BAZ1A and SCML1 continue to display a mutually exclusive pattern of expression, whereas in (F) cKO SCML1 is found accumulated in the sex body alongside with BAZ1A (yellow). Colocalization of SCML1 and BAZ1A in *Dicer1* cKO was confirmed by ImageJ: red line BAZ1A, green line SCML1; and quantified manually in the sex bodies in stage IX–X (*n* = 129 and *n* = 133 for WT and *Dicer1* cKO, respectively). Basement membrane of seminiferous epithelium is marked with a dotted line. Insets 1 (A, B, C and D) and 1–3 (E and F) are derived from boxed areas. Scale bars 10 µm.

**Figure 7:**
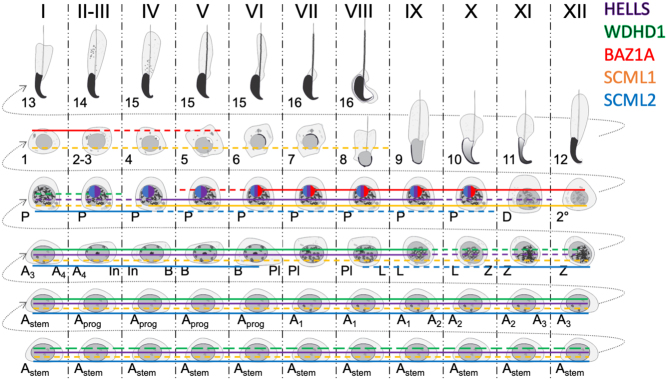
Summary of spermatogenic expression pattern of the proteins analyzed in this study. The expression of HELLS (purple), WDHD1 (green), BAZ1A (red), SCML1 (orange) and SCML2 (blue) in different spermatogenic cell types. Solid line, high expression level. Dashed line, low expression level. Sex body (sphere in pachytene spermatocytes) in stage II–V pachytene spermatocytes stains positively for SCML2 (blue) and HELLS (purple). In stages VI–X ,SCML2 (blue), HELLS (purple) and BAZ1A (red) colocalize in the sex body in WT mouse testis.

In our previous study, we showed that *Dicer1* cKO testes lack small RNAs derived from MSR forward transcripts, enriched in nuclear fractions (Yadav *et al.* 2020). Thus, this reduction in nuclear small RNAs prompted us to investigate the localization of another Polycomb protein family member, SCML1. SCML1 has been earlier reported to be a component of meiotic dense body (Papanikos *et al.* 2017) that is associated with the sex body and enriched with small non-coding RNAs including miRNAs and PIWI-interacting RNAs (piRNAs). To analyze this interesting finding in more detail, we performed co-localization studies of SCML1 with respect to BAZ1A as a sex body marker (Fig. 6). Interestingly, we showed that in WT testis, SCML1 formed distinct nuclear foci in spermatocytes that did not overlap with BAZ1A-positive sex bodies but were frequently closely associated with them. However, in *Dicer*1-cKO spermatocytes, the organization of SCML1-positive foci was disrupted and the SCML1 signal spread to the sex body, most pronouncedly in mid-to-late epithelial stages (Fig. 6), suggesting that regulation of the sex body heterochromatin is affected in the absence of DICER. Interestingly, in WT testis SCML1 was found in complex with DICER (Fig. 5D and E) suggesting that they function together in distinct aspects of chromatin regulation. A summary of the expression patterns of the genes and proteins analyzed in this study is provided in Fig. 7 and Supplementary Fig. 2B, respectively.

## Discussion

Heterochromatin is a key chromosomal domain, and its dysregulation in the germline is accompanied with chromosomal missegregation, aneuploidy and meiotic failure (Peters *et al.* 2001, Tachibana *et al.* 2007, Yadav *et al.* 2020, Cheng *et al.* 2021). To better understand the regulation of heterochromatin in male germ cells, we characterized the expression of chromatin modifiers HELLS, WDHD1 and BAZ1A and showed that they display distinct, dynamic patterns of expression during spermatogenesis and are thus likely to regulate different aspects of heterochromatin biology. We have previously shown that PCH-derived MSR transcripts are regulated by DICER, and DICER is also involved in the association of SET-domain containing methyltransferases with heterochromatin (Yadav *et al.* 2020). Here we show that HELLS and BAZ1A, but not WDHD1, interact with both DICER and MSR DNA, making them potentially relevant partners of DICER in regulation of MSR chromatin and PCH stability in the male germline. The interaction of DICER with different chromatin remodelers in male germ cells emphasizes its nuclear roles that go beyond its canonical RNAi functions (Kurzynska-Kokorniak *et al.* 2015, Song & Rossi 2017). This chromatin-related function is further supported by our finding that sex body organization is affected in the absence of DICER.

Testis is one of the few adult tissues expressing high levels of HELLS (Geiman *et al.* 1998), a protein that has been shown to modulate the establishment of DNA methylation patterns during cellular differentiation and to regulate chromatin accessibility (Huang *et al.* 2004, Dunican *et al.* 2013, Yu *et al.* 2014, Ren *et al.* 2015). We found that HELLS is expressed by most premeiotic germ cells, including A_undiff_ spermatogonia, and by spermatocytes up to late pachynema, where it enriches in the sex body of mid-to-late pachytene spermatocytes. HELLS has been shown to be required for normal spermatogenesis, and adult *Hells*-cKO mice exhibit degeneration of the seminiferous epithelium (Spruce *et al.* 2020). *Hells* deficiency also results in a meiotic arrest at early to mid-pachynema, spermatocyte apoptosis and reduced proliferation of spermatogonia (Zeng *et al.* 2011, Spruce *et al.* 2020). The potential role of HELLS in A_undiff_ is interesting not only because it has been proposed to play a role in embryonic stem (ES) cell and neural stem cell differentiation and self-renewal (Xi *et al.* 2009, Han *et al.* 2017), which share some molecular resemblance with A_undiff_ but also because *Hells* mutant mice display growth retardation and premature aging, suggesting a more general stem cell defect associated with improper HELLS action (Sun *et al.* 2004). Considering our earlier findings of DICER-mediated processing of MSR transcripts at PCH (Yadav *et al.* 2020), as well as hypomethylation of repetitive sequences in DICER-deficient ES cells (Kanellopoulou *et al.* 2005), it is an intriguing hypothesis that RNAi machinery and HELLS might be integrated at repetitive sequences in the male germline to control DNA methylation and heterochromatin maintenance.

BAZ1A is also highly expressed in the testis, and while BAZ1A seems to be dispensable for development and viability of embryos and adults, spermatogenesis is severely affected by the loss of *Baz1a* (Jones *et al.* 2000, Dowdle *et al.* 2013).* Baz1a*-deficient male mice display pronounced spermiogenic defects and misregulation of meiotic and postmeiotic gene expression programs (Dowdle *et al.* 2013). We showed here that BAZ1A localizes to the sex body and PCH in spermatocytes, and our ChIP assay demonstrated the association with many repeat sequences, including PCH. The interaction of BAZ1A and DICER suggests that they could act together on chromatin. Given the well-known function of BAZ1A as a part of multiprotein chromatin remodeler complexes ACF and CHRAC (Morgan *et al.* 2020), it is tempting to speculate that BAZ1A could co-operate with DICER-mediated RNAi to regulate repeat-containing chromatin through its nucleosome remodeling activity, for example by promoting PCH transcription.

WDHD1 has been previously shown to be involved in epigenetic and transcriptional regulation of MSR chromatin in cultured somatic cells (Hsieh *et al.* 2011). In these cells, downregulation of WDHD1 results in destabilization of the epigenetic status of heterochromatin and defective mitosis and also leads to increased levels of MSR transcripts, bearing a striking resemblance to *Dicer1* cKO testis (Hsieh *et al.* 2011, Yadav *et al.* 2020). Considering these data, it is surprising that we did not see the association of WDHD1 with MSR sequences nor DICER in the male germline. The reason for the discrepancy might be that in the previous study, WDHD1-MSR interaction was studied *in vitro* (Hsieh *et al.* 2011), whereas we used an *in vivo* approach. Importantly, among all human adult tissues, WDHD1 is exclusively expressed in the testis (Sato *et al.* 2010). Nevertheless, the expression pattern of WDHD1 during mouse spermatogenesis is intriguing. The highest levels were seen in differentiating spermatogonia and early spermatocytes. In A_undiff,_ the expression was inversely related to stemness with gradually increasing levels as A_undiff_ progressed A_s_ -> A_pr_ -> A_al4_ and from GFRa1-positive to SOX3-positive state. A number of other genes and proteins, including SOX3, *Ngn3*, RARγ (retinoic acid receptor gamma), *Pou5f1* (POU Class 5 Homeobox 1), LIN28 and *Piwil4* (Piwi-like protein 4), have been shown to behave in a similar way (Nakagawa *et al.* 2007, Ikami *et al.* 2015, Carrieri *et al.* 2017, La *et al.* 2018, McAninch *et al.* 2020). Altogether, these findings suggest that WDHD1 might play a role in the spermatogenic differentiation commitment and future studies are warranted to investigate its role in this significant developmental shift.

BAZ1A and HELLS, which both were shown to associate with DICER and the MSR DNA, were also both accumulated in the sex body. Although the localization of HELLS and BAZ1A to the sex body was not affected in *Dicer1* cKO spermatocytes, the sex body organization was affected, as visualized by disperse SCML1 localization. There are limited data about SCML1, a novel gametogenesis and meiosis-associated protein, shown to be dispensable for mouse spermatogenesis but associated with azoospermia in human (Papanikos *et al.* 2017, Milunsky *et al.* 2020).Papanikos *et al.* (2017) have recently shown that SCML1 expression is restricted to the meiotic dense body, an enigmatic chromatin-free nuclear structure enriched with small-RNAs, and proposed to play a role in sex body formation (Papanikos *et al.* 2017). The localization pattern we observed was somewhat different, with several nuclear SCML1-positive patches in most seminiferous epithelial cells, particularly in pachytene spermatocytes and round spermatids. Notably, the SCML1-foci are generally located in euchromatic regions; a finding that would also be supported by a transcription factor function previously assigned to SCML1 (Nan *et al.* 2019). While the physiological significance of the partial spreading of SCML1 to the sex body domain upon *Dicer1* ablation awaits further elucidation, it suggests that SCML1 is involved in the coordination of sex body organization. It also suggests that DICER activity is required for the regulation of sex chromosomes during meiotic division, a hypothesis also supported by the affected sex chromosome integrity and the misregulation of sex chromosomal genes in *Dicer1* knockout animals (Greenlee *et al.* 2012, Modzelewski *et al.* 2015). Our findings lay the basis for a more in-depth study of these proteins, interactions and modes of function in the chromatin regulation in the mouse germline.

## Supplementary Material

Figure S1. Localization of HELLS and HP1β in the mouse testis. A)

Fig. S2. Reanalysis of published spermatogenic single-cell RNA-seq data. A)

Fig. S3. SCML2 colocalizes with BAZ1A in the sex body of mid-to-late pachytene spermatocytes.

## Declaration of interest

Noora Kotaja is an Associate Editor of *Reproduction*. Noora Kotaja was not involved in the review or editorial process for this paper, on which she is listed as an author. The other authors declare no conflict of interest.

## Funding

This work was supported by the Sigrid Jusélius Foundation, the Academy of Finland http://dx.doi.org/10.13039/501100002341, the Novo Nordisk http://dx.doi.org/10.13039/501100004191 Foundation and the Jalmari and Rauha Ahokas Foundation.

## Author contribution statement

R P Y conceived the study, performed experiments, analysed data and edited the manuscript. S L performed experiments and analysed data. L M performed bioinformatics analyses on scRNA-seq data. J-A M performed experiments, analysed data and wrote the manuscript. N K conceived the study and wrote the manuscript. J-A M and N K contributed equally to this work
