# Supplementary figures and images for: Chromatin remodelers HELLS, WDHD1 and BAZ1A are dynamically expressed during mouse spermatogenesis
Source: Reproduction. 2022 Oct 4;165(1):49–63. doi: 10.1530/REP-22-0240 (PMC9782464; doi:10.1530/REP-22-0240)

Figure S1

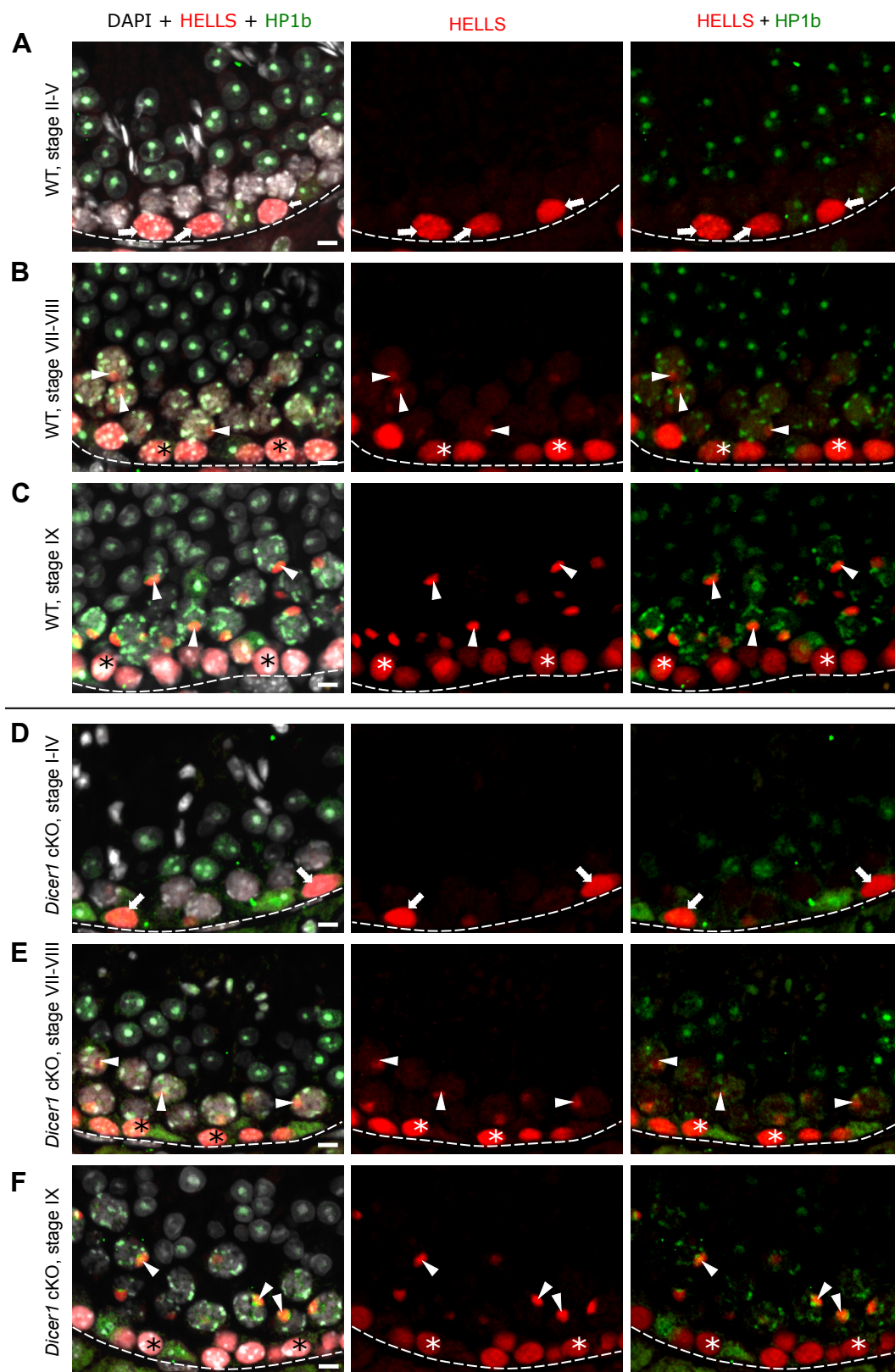

Supplement: Figure S1. Localization of HELLS and HP1β in the mouse testis. A) [file supplementary_figure_1.pdf]

Figure S2

A

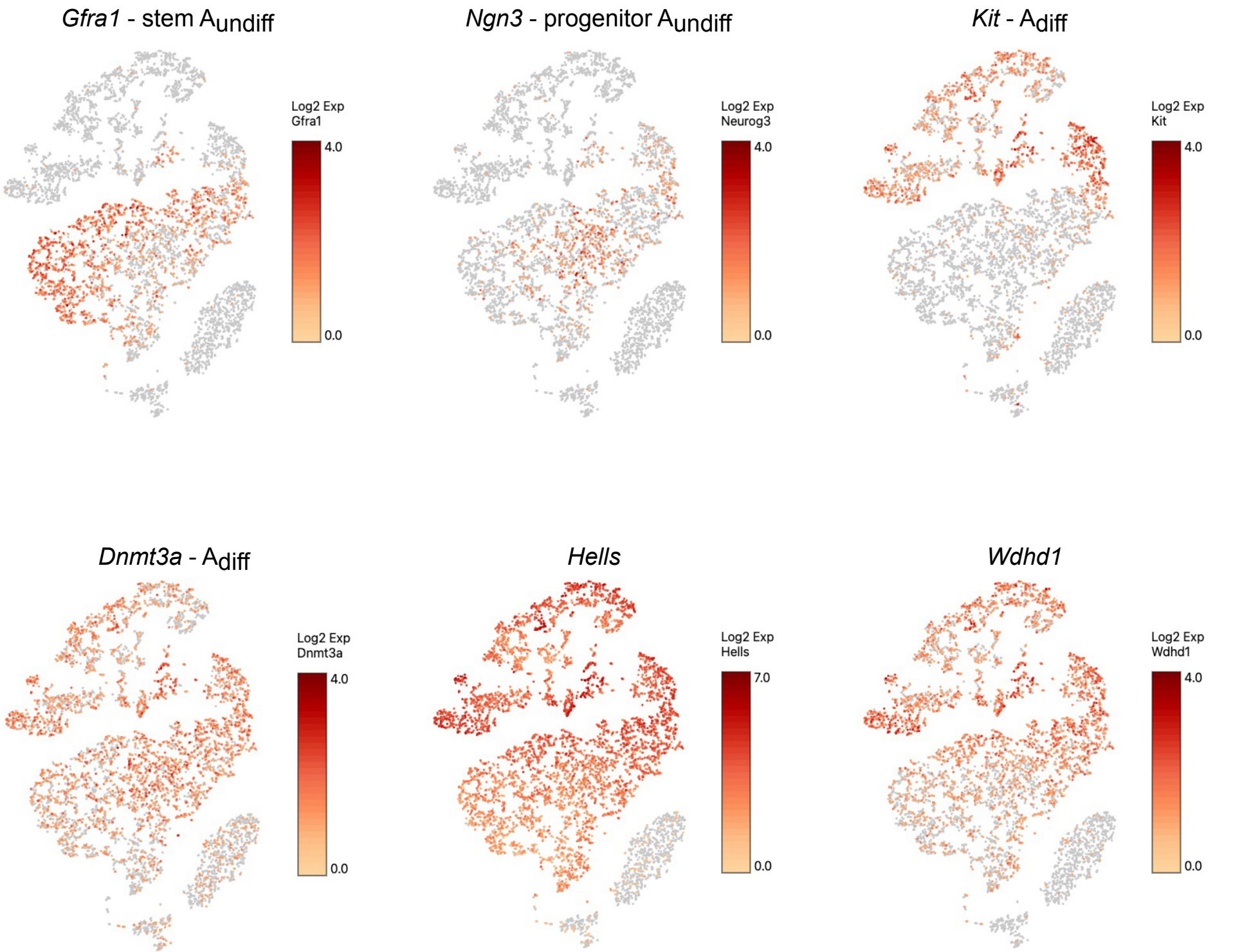

B

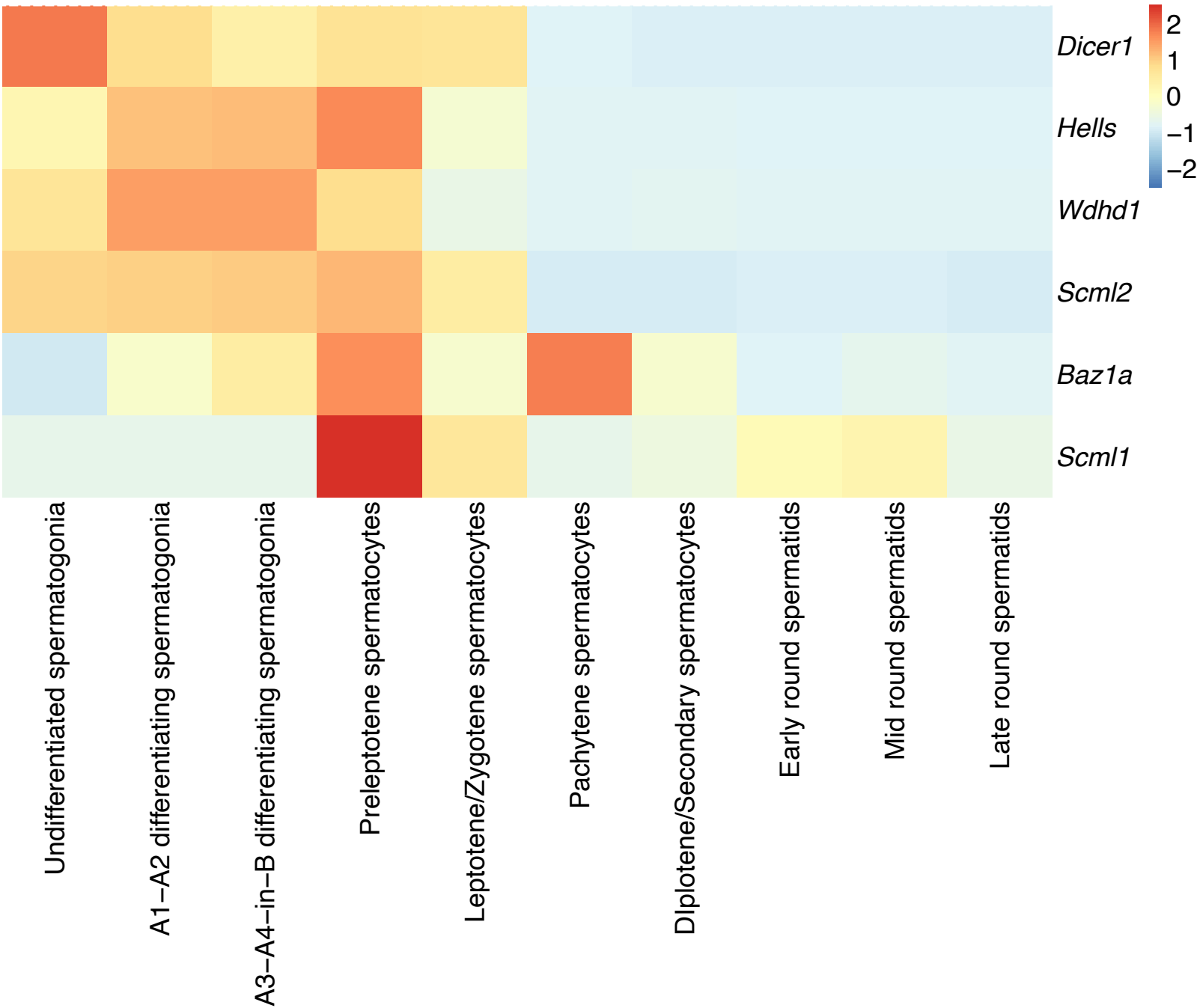

Supplement: Fig. S2. Reanalysis of published spermatogenic single-cell RNA-seq data. A) [file supplementary_figure_2.pdf]

Figure S2

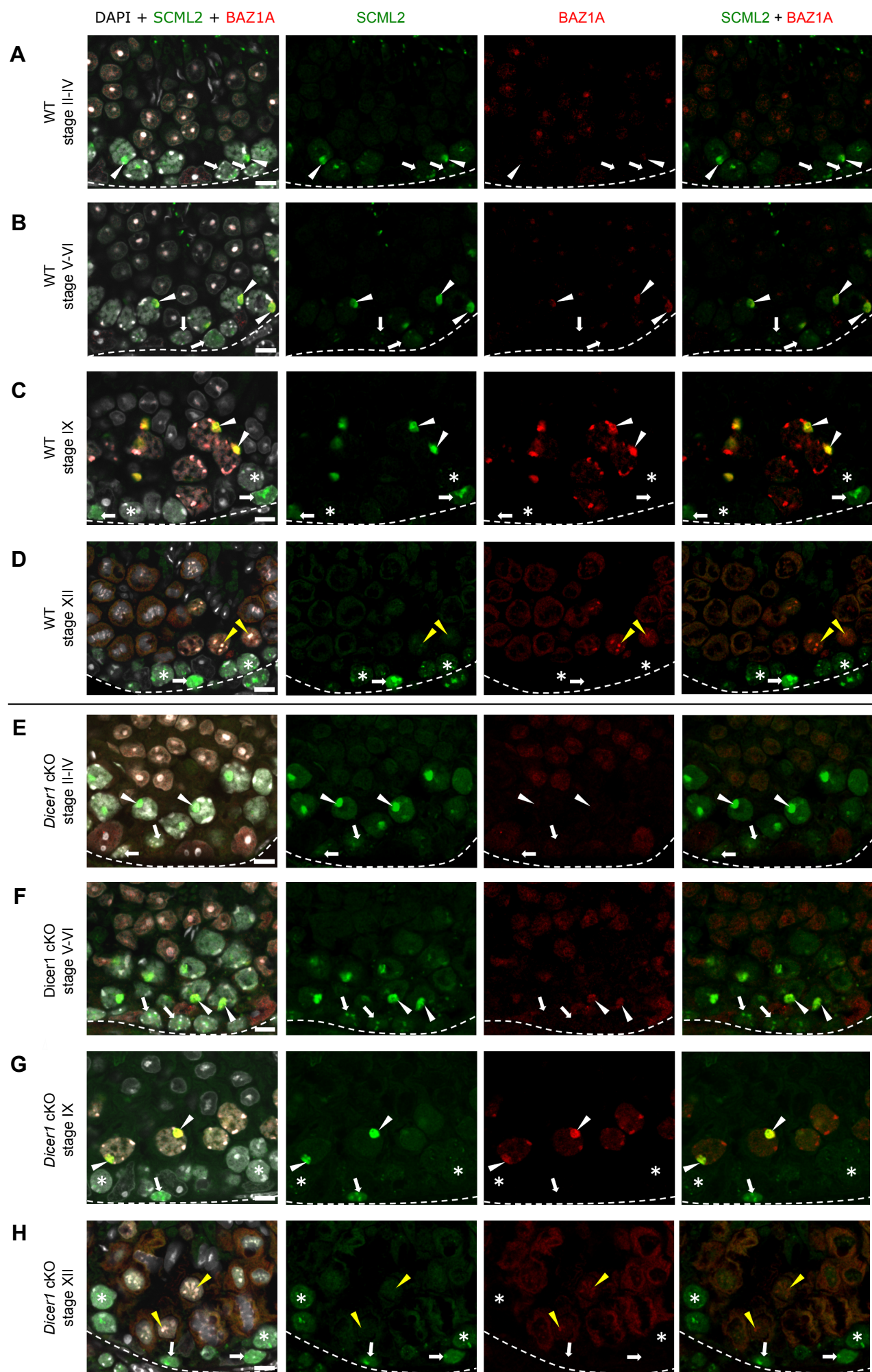

Supplement: Fig. S3. SCML2 colocalizes with BAZ1A in the sex body of mid-to-late pachytene spermatocytes. [file supplementary_figure_3.pdf]
